# Severe Metabolic Decompensation in Metastatic Sarcomatoid Renal Cell Carcinoma During Immune Checkpoint Inhibitor Therapy: A Case Report and Literature Review

**DOI:** 10.3390/diagnostics16111679

**Published:** 2026-05-29

**Authors:** Lorena Ciumărnean, Cezara Andreea Gerdanovics, Olga Hilda Orășan, Alexandru Gerdanovics, Nicoleta Valentina Leach, Ioana Para, Gabriela Bombonica Dogaru

**Affiliations:** 1 Department 2, Faculty of Nursing and Health Sciences, “Iuliu Hațieganu” University of Medicine and Pharmacy Cluj-Napoca, Republicii Street, No. 18, 400015 Cluj-Napoca, Romanianicoleta_leach@yahoo.com (N.V.L.); 2 Department of Internal Medicine, 4 th Medical Discipline, “Iuliu Hațieganu” University of Medicine and Pharmacy Cluj-Napoca, Republicii Street, No. 18, 400015 Cluj-Napoca, Romania; 3 Department of Pathophysiology, “Iuliu Haţieganu” University of Medicine and Pharmacy Cluj-Napoca, Victor Babeş Street, No. 2-4, 400012 Cluj-Napoca, Romania; alexandru.gerdanovics@elearn.umfcluj.ro; 4 Clinical Rehabilitation Hospital, Viilor Street, No. 46-50, 400066 Cluj-Napoca, Romania; 5 Department of Balneophysiokinetotherapy and Medical Recovery, Faculty of Nursing and Health Sciences, “Iuliu Hațieganu” University of Medicine and Pharmacy Cluj-Napoca, Victor Babeş Street, No. 8, 400347 Cluj-Napoca, Romania

**Keywords:** sarcomatoid renal cell carcinoma, immune checkpoint inhibitors, immune-related adverse events, hypophysitis, hypothyroidism, hyperkalemia, acute kidney injury, endocrine toxicity, metabolic decompensation

## Abstract

**Background and Clinical Significance**: Sarcomatoid renal cell carcinoma is a rare and highly aggressive variant of renal cell carcinoma, frequently associated with advanced-stage disease, early metastatic spread, and poor prognosis. Although immune checkpoint inhibitors have improved outcomes in metastatic renal cell carcinoma, particularly in tumors with sarcomatoid differentiation, they may also induce severe immune-related adverse events involving multiple organ systems. **Case Presentation:** We report the case of a 54-year-old woman with metastatic clear cell renal cell carcinoma with sarcomatoid differentiation, previously treated with nivolumab plus ipilimumab and subsequently with pazopanib, who was admitted with severe dehydration, repeated vomiting, marked asthenia, lower-limb-predominant muscle weakness, and inability to maintain orthostatism. Laboratory investigations revealed severe hyperkalemia, hyponatremia, hypoglycemia, anemia, thrombocytopenia, and prerenal acute kidney injury. The patient had a previous history of severe endocrine immune-related toxicity, including autoimmune hypophysitis and hypothyroidism, which had led to discontinuation of immunotherapy. Following fluid resuscitation, electrolyte correction, and supportive treatment, the metabolic abnormalities resolved and renal function improved significantly. Given the severity of the muscle weakness, a possible immune-mediated neuromuscular adverse event was also considered, although hyperkalemia remained a plausible contributing factor. **Conclusions**: This case highlights the complex interplay between prior immune checkpoint inhibitor exposure, endocrine dysfunction, metabolic decompensation, and possible neuromuscular involvement in metastatic sarcomatoid renal cell carcinoma. Early recognition, careful differential diagnosis, and multidisciplinary management are essential to prevent rapid clinical deterioration and optimize outcomes in patients with complex immune-related toxicities.

## 1. Introduction

Renal cancer represents a significant global health burden. In the United States alone, approximately 73,750 new cases were estimated to be diagnosed in 2020, accounting for nearly 14,830 deaths [1]. Renal cell carcinoma (RCC) accounts for nearly 90% of all malignancies arising from the renal parenchyma [2].

A rare but clinically significant phenomenon, known as sarcomatoid dedifferentiation, may occur across most histological subtypes of RCC and is associated with particularly aggressive tumor behavior and poor prognosis. Tumors exhibiting this transformation are commonly referred to as sarcomatoid renal cell carcinomas (sRCCs). Patients with sRCC frequently present with advanced or metastatic disease, and survival beyond one year is uncommon [1]. Sarcomatoid features are identified in approximately 4–5% of all RCC cases [3], although the reported prevalence varies considerably, ranging from 1% to 29%, depending on the primary histological subtype and the study population [4]. Although sRCC is less commonly diagnosed in localized disease, accounting for approximately 20–40% of cases, nearly 20% of patients with metastatic RCC exhibit sarcomatoid dedifferentiation [5].

Sarcomatoid RCC is most frequently diagnosed between the ages of 54 and 63 years, with a male-to-female ratio ranging from 1.3:1 to 2:1 [6]. The mechanisms underlying this gender disparity remain unclear; however, possible explanations include historical differences in occupational exposures or smoking habits, as well as the potential influence of sex hormones on tumor biology [7]. The natural history of sarcomatoid RCC is particularly unfavorable, as approximately 60–80% of patients present with advanced or late-stage disease at diagnosis.

Until recently, the median overall survival of patients with sarcomatoid renal cell carcinoma (sRCC) was approximately 6–13 months [8,9]. However, the emergence of combination immune checkpoint inhibitor (ICI) therapy, particularly nivolumab (NIVO; a programmed death-1 immune checkpoint inhibitor antibody) combined with ipilimumab (IPI; a cytotoxic T-lymphocyte antigen-4 antibody), as a first-line treatment for advanced clear cell RCC has substantially improved survival outcomes in patients with advanced disease [10].

A post hoc analysis of the phase III CheckMate 214 trial further demonstrated promising efficacy of the nivolumab–ipilimumab combination in patients with sRCC compared with sunitinib, with a median overall survival of 31.2 months versus 13.6 months and an overall response rate (ORR) of 57% versus 19%, respectively [11].

Despite these therapeutic advances, immune checkpoint inhibitors are associated with a unique spectrum of immune-related adverse events (irAEs), which may lead to significant morbidity due to autoimmune-like toxicities [12]. Although irAEs have been extensively described in several solid tumors, the safety profile of combined nivolumab and ipilimumab therapy in renal cell carcinoma is still being fully characterized.

These therapies have significantly improved both progression-free survival (PFS) and overall survival (OS), establishing a new standard of care for metastatic RCC [11,13]. Consequently, although the incidence of RCC has been increasing globally, mortality rates have shown a gradual decline [14]. In particular, for the treatment of metastatic RCC (mRCC), dual immune checkpoint blockade targeting cytotoxic T-lymphocyte-associated antigen-4 (CTLA-4) and programmed cell death-1 (PD-1) pathways (e.g., ipilimumab plus nivolumab), as well as combinations of anti-PD-1 ICIs with vascular endothelial growth factor (VEGF) tyrosine kinase inhibitors (e.g., nivolumab plus cabozantinib, pembrolizumab plus axitinib, or pembrolizumab plus lenvatinib), have been approved as first-line systemic therapies [13,15].

Although the efficacy of ICIs in metastatic RCC is well established, their use has been associated with a broad spectrum of immune-related adverse events affecting multiple organ systems, including the endocrine system [16]. Endocrine toxicities are relatively common and may involve the thyroid, parathyroid glands, pituitary gland, adrenal glands, and pancreas, leading to conditions such as hypothyroidism, hyperthyroidism, thyroid eye disease, hypoparathyroidism, hypophysitis, adrenal insufficiency, and diabetes mellitus [17,18].

While endocrine irAEs most frequently occur within 3–6 weeks after the initiation of therapy, they may develop at any time during treatment or even after treatment discontinuation [19,20]. Recent studies suggest that endocrine toxicities may be irreversible in approximately 50% of cases [17] and can become life-threatening if not promptly recognized and appropriately managed. Delayed diagnosis is not uncommon and may ultimately require discontinuation of immune checkpoint inhibitor therapy [12].

This case illustrates the complex interaction between advanced malignancy, systemic oncologic therapy, endocrine dysfunction, and metabolic complications in sarcomatoid renal cell carcinoma. In line with current literature, immune checkpoint inhibitors, while improving survival, are associated with immune-related endocrine toxicities that may contribute to metabolic instability. The objective of this review is to illustrate this interaction and to emphasize the importance of early recognition and multidisciplinary management of endocrine and metabolic complications in order to prevent clinical deterioration and allow continuation of oncologic therapy.

## 2. Case Presentation

### 2.1. General Data of the Patient, Oncologic Evolution and Treatment

We present the case of a 54-year-old female with a history of right-sided clear cell renal cell carcinoma with sarcomatoid differentiation, status post nephrectomy, staged as pT3aNxMx, Fuhrman grade 4, WHO grade 4, with lymphovascular invasion (L1V1) and perirenal extension.

The perifascial nephrectomy specimen consisted of a tumoral kidney measuring 13 cm × 11 cm × 7.5 cm with attached perirenal adipose tissue. Gross examination revealed a heterogeneous renal tumor measuring 8.5 cm × 9 cm × 7 cm, composed of a yellow-golden area and a whitish firm area. The renal vein was free of tumor involvement.

**Figure 1:**
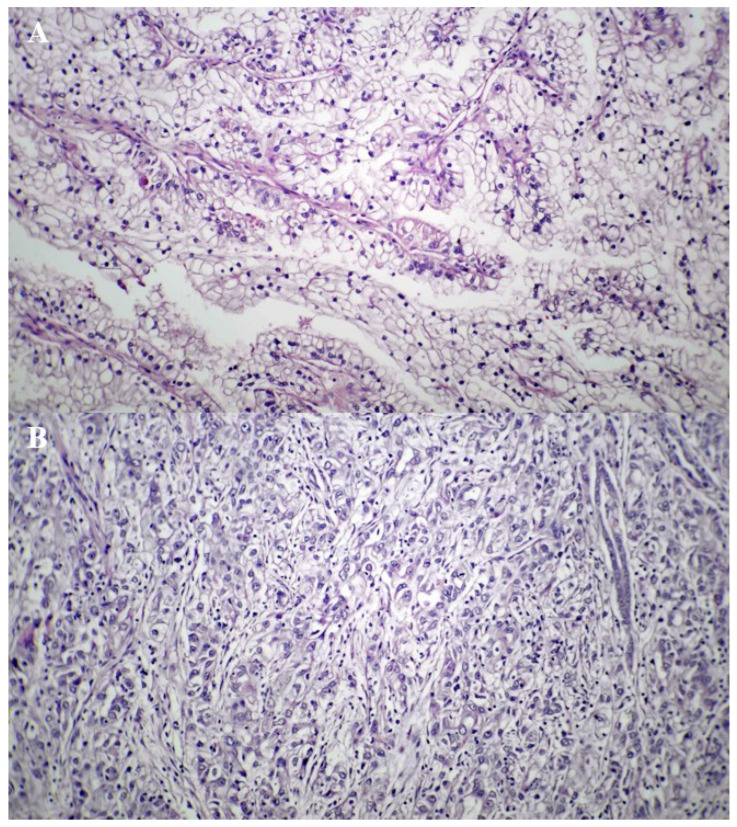
Histopathological features of clear cell renal cell carcinoma with sarcomatoid differentiation. (**A**) Hematoxylin and eosin staining showing the conventional clear cell renal cell carcinoma component, composed of tumor cells with optically clear cytoplasm arranged in nests/alveolar structures separated by a delicate vascular network. (**B**) Hematoxylin and eosin staining showing the sarcomatoid component, characterized by high-grade spindle and pleomorphic tumor cells embedded in a desmoplastic stroma, with loss of the conventional clear cell architecture. The overall findings supported the diagnosis of clear cell renal cell carcinoma with sarcomatoid differentiation, corresponding to Fuhrman grade 4/WHO/ISUP grade 4. Original magnification: ×10.

Histopathological examination demonstrated clear cell renal cell carcinoma with renal sinus invasion and venous invasion. The whitish tumor areas showed tubular architecture and intratumoral desmoplasia, initially raising the possibility of a collecting duct carcinoma component. However, immunohistochemistry was negative for GATA3, 34βE12, and p63 in both tumor components, while PAX8 was non-contributory. CD10 was diffusely and strongly positive, p504s was weakly positive in the suspected collecting duct-like area, and CK7 showed only focal positivity in both components. The CK7-positive tubular structures were interpreted as residual renal tubules distorted by tumor invasion. These findings supported the final diagnosis of clear cell renal cell carcinoma with sarcomatoid differentiation, classified as Fuhrman grade 4/WHO/ISUP grade 4 (Figure 1).

The histopathological assessment was performed approximately two years earlier in an external pathology department and the final report available to our team established the diagnosis of clear cell renal cell carcinoma with sarcomatoid differentiation, WHO/ISUP grade 4. However, the percentage of the sarcomatoid component within the overall tumor volume was not quantified in the original pathology report.

The disease subsequently evolved with metastatic involvement of the lungs and the contralateral kidney. The patient was classified as high risk according to the IMDC score and was initially treated with combination immunotherapy consisting of nivolumab and ipilimumab, followed by tyrosine kinase inhibitor therapy with pazopanib.

Her past medical history was notable for severe hypothyroidism following total thyroidectomy for benign disease, essential hypertension grade I, silent myocardial ischemia, moderate mitral regurgitation, mild aortic regurgitation, moderate tricuspid regurgitation, and chronic heart failure (NYHA class II) with preserved left ventricular ejection fraction (LVEF 55%).

The oncologic course began with right nephrectomy performed with curative intent. Histopathological examination confirmed a high-grade tumor (WHO grade 4) with sarcomatoid differentiation and staging of pT3aNxMx. During further evaluation for adjuvant pembrolizumab therapy, imaging revealed disease progression with the development of pulmonary and contralateral renal metastases. Given the high-risk classification according to IMDC criteria, combination immunotherapy with nivolumab and ipilimumab was initiated.

The induction phase consisted of four treatment cycles administered between December 2024 and January 2025, followed by maintenance therapy with nivolumab. Initial imaging evaluation was favorable, with thoraco-abdomino-pelvic CT suggesting tumor remission.

In June 2025, maintenance immunotherapy was discontinued due to severe immune-related toxicity, characterized by significant endocrine dysfunction, including autoimmune hypophysitis and severe hypothyroidism. The patient required corticosteroid therapy, endocrinological evaluation, and adjustment of thyroid hormone replacement.

Subsequently, in October 2025, disease progression with bone involvement was documented, and second-line therapy with the tyrosine kinase inhibitor pazopanib was initiated, in association with antiresorptive treatment. The clinical course under this regimen was fluctuating, requiring therapeutic adjustments due to cardiovascular and endocrine comorbidities, although an overall clinical benefit was maintained. Pazopanib had been discontinued approximately two weeks before the current admission in January 2026. Therefore, the patient was not receiving pazopanib at the time of hospitalization.

### 2.2. Actual Presentation

The current hospitalization occurred in the context of severe dehydration, metabolic disturbances, and prerenal acute kidney injury. These complications developed against the background of complex oncologic evolution and ongoing systemic therapy. The patient presented to the Emergency Department of the Municipal Hospital of Cluj-Napoca with severe dehydration, nausea, repeated vomiting, and marked asthenia, with an insidious onset approximately two days before presentation. She also reported muscle weakness predominantly affecting the lower limbs and myalgia, stating that she was unable to maintain an upright standing position. Initial laboratory evaluation revealed severe hyperkalemia (6.8 mEq/L), hypoglycemia, anemia, and mild thrombocytopenia. She was subsequently transferred and admitted to the Internal Medicine Department of the Clinical CF Hospital in Cluj-Napoca for further investigations and specialized treatment. Family history was unremarkable for the current pathology. The patient was retired and denied smoking or alcohol consumption.

Chronic home medication prior to admission included levothyroxine (Euthyrox) 175 µg (morning administration), dapagliflozin 10 mg once daily, acetylsalicylic acid 75 mg once daily, rosuvastatin 10 mg once daily, sacubitril/valsartan 24/26 mg twice daily, calcium and vitamin D supplementation, and opioid analgesia (Sevredol) as needed, which had been discontinued approximately two weeks prior to admission.

On physical examination at admission, the patient appeared severely ill but was conscious and oriented. She was cachectic, with reported weight loss in recent months (exact weight unknown). The skin and mucous membranes were pale and dehydrated. A postoperative scar was present on the right flank. Hair and nails were normal. The subcutaneous adipose tissue was poorly represented, and no superficial lymphadenopathy was detected. The muscular system was hypotonic and hypokinetic. The osteoarticular examination revealed pain in the right knee, persistent muscle weakness predominantly affecting the lower limbs, and generalized myalgia, all of which had limited the patient’s mobility over the preceding two weeks.

Pulmonary examination revealed normal percussion, preserved tactile fremitus, and bilateral vesicular breath sounds without added sounds. Cardiovascular examination showed rhythmic heart sounds, synchronous with the peripheral pulse, without audible murmurs. Peripheral pulses were palpable bilaterally at the dorsalis pedis arteries. Blood pressure at admission was 90/69 mmHg, and heart rate was 94 bpm.

The abdomen was soft, non-tender, and mobile with respiration, without palpable masses. The patient reported absence of bowel movements for at least 10 days. The liver was palpable on deep inspiration, while the spleen was not palpable. The right renal lodge was surgically absent, renal fossae were otherwise unremarkable, and Giordano’s sign was negative bilaterally. Diuresis was reduced. Neurological examination was unremarkable, with preserved temporal and spatial orientation and no signs of focal deficit or meningeal irritation.

Electrocardiography showed sinus rhythm with a ventricular rate of 95 bpm, intermediate electrical axis, no ST-segment changes, and T-wave inversion in the anterolateral leads. Echocardiography revealed a slightly hyperechogenic pericardium, preserved systolic function (LVEF 55%), moderate mitral regurgitation, mild aortic regurgitation, and moderate tricuspid regurgitation.

Laboratory evaluation (Table 1) demonstrated hyperkalemia, hyponatremia, inflammatory syndrome, anemia, leukopenia, thrombocytopenia, mild hypocalcemia (likely secondary to hemodilution following fluid resuscitation), hypoglycemia, azotemia with reduced estimated glomerular filtration rate, hyperuricemia, and elevated NT-proBNP, myoglobin, D-dimer, and ferritin levels, as well as iatrogenic hyperthyroidism.

During hospitalization, treatment was initiated for hydro-electrolytic rebalancing and correction of hyperkalemia, including administration of rapid-acting insulin with 10% glucose and calcium gluconate. Additional therapies included antiemetics, gastric antisecretory agents, analgesics, diuretics, and venous thromboembolism prophylaxis. Thyroid hormone replacement therapy was temporarily discontinued.

Under treatment, hydration was gradually restored, followed by resumption of oral intake. Bowel transit resumed, initially with hard stools and subsequently normalizing. Diuresis improved and became normal to mildly increased. In the context of progressively decreasing hemoglobin levels, intravenous iron (ferric carboxymaltose—Ferinject) and a dose of human albumin were administered. Antihypertensive therapy was also adjusted.

During the course of hospitalization, hydro-electrolytic disturbances resolved and renal function improved, with stabilization of laboratory parameters and significant clinical improvement.

Given the vomiting syndrome, muscle weakness predominantly affecting the lower limbs, and the oncological history, the possibility of brain metastases was considered, as these are relatively frequent in this type of malignancy and could have explained the vomiting syndrome. In this context, the patient was referred by the oncologist for a brain CT scan. The investigation was subsequently performed at the Cluj-Napoca County Emergency Clinical Hospital and did not reveal any lesions suggestive of secondary brain involvement.

In addition, from the time of admission, the patient presented with severe generalized muscle weakness, with loss of the ability to walk. Although hydro-electrolytic disturbances resolved and renal function improved after treatment, the muscle weakness did not completely remit following correction of hyperkalemia. This persistence of severe weakness, particularly with loss of ambulation, made isolated hyperkalemia-induced paralysis less likely and supported the suspicion of an immune-mediated neuromuscular adverse event, including possible myasthenia gravis. Therefore, further neurological evaluation, including electroneuromyography, was recommended.

Based on the clinical, laboratory, and imaging findings, the following diagnoses were established: severe dehydration syndrome, medically corrected hyperkalemia, prerenal acute kidney injury, metastatic clear cell renal cell carcinoma with sarcomatoid differentiation (pT3aNxMx, Fuhrman grade 4), essential hypertension grade I, silent myocardial ischemia, multiple valvular insufficiencies (mitral grade II, aortic grade I, tricuspid grade II), chronic heart failure NYHA class II with preserved ejection fraction, normocytic normochromic anemia likely secondary to iron deficiency, and severe hypothyroidism following total thyroidectomy.

The patient was discharged in improved condition with recommendations for continuation of chronic therapy, endocrinological reassessment for thyroid hormone replacement adjustment, ongoing oncologic and cardiologic follow-up, and outpatient evaluation within 2–3 weeks or as needed.

The diagnosis of an immune-mediated neuromuscular adverse event could not be confirmed during hospitalization. Antibody testing, including anti-acetylcholine receptor, anti-MuSK, and anti-LRP4 antibodies, was not available in our institution and was therefore recommended to be performed in an outpatient neurological setting. Electroneuromyography and further neurophysiological testing were also scheduled after discharge. However, the patient’s clinical condition subsequently deteriorated, and she was unable to attend the planned neurological investigations. Therefore, myasthenia gravis remained a clinical suspicion rather than a confirmed diagnosis.

## 3. Discussion

### 3.1. Sarcomatoid Renal Cell Carcinoma

Renal cell carcinoma (RCC) with sarcomatoid dedifferentiation (sRCC) may arise from any histological subtype of RCC [21]. Compared to non-sarcomatoid RCC, this variant is associated with significantly higher cancer-specific and overall mortality across all disease stages [5]. A considerable proportion of patients with sRCC present with synchronous metastatic disease at diagnosis, and up to 20% of metastatic RCC cases exhibit sarcomatoid dedifferentiation. In line with these observations, our patient presented with an aggressive disease course characterized by early metastatic involvement of the lungs and contralateral kidney, reflecting the high metastatic potential and unfavorable prognosis associated with sarcomatoid differentiation.

In 2022, the World Health Organization (WHO) and the International Society of Urological Pathology (ISUP) introduced an updated four-tier grading system, replacing the traditional Fuhrman classification [22]. Advances in high-throughput molecular analyses have demonstrated distinct grade-dependent molecular and metabolic profiles in clear cell RCC (ccRCC), highlighting their potential as therapeutic targets [23,24].

Sarcomatoid features are no longer considered a distinct histological subtype of RCC. Instead, sarcomatoid dedifferentiation represents a high-grade transformation that may arise within a wide spectrum of established RCC histologies, including clear cell, papillary, and chromophobe RCC, as well as rarer entities such as collecting duct carcinoma, thyroid-like follicular carcinoma, acquired cystic disease–associated RCC, mucinous tubular and spindle cell carcinoma, ALK-rearranged RCC, succinate dehydrogenase–deficient RCC, and malignant mixed epithelial and stromal tumors [25]. Among these, clear cell RCC and chromophobe RCC are the most frequently associated with sarcomatoid differentiation. In the present case, sarcomatoid dedifferentiation occurred within a clear cell RCC, consistent with the most commonly reported histological background, and likely contributed to the aggressive clinical course observed.

The clinical presentation of sarcomatoid renal cell carcinoma (sRCC) is highly variable and largely dependent on the stage of disease at diagnosis. Most patients present with locally advanced or metastatic disease, with up to 90% being symptomatic at the time of diagnosis. Clinical manifestations are typically nonspecific and may include flank or abdominal pain (reported in approximately 51% of symptomatic patients), hematuria (22–34.7%), weight loss (18–22.6%), fatigue (15%), fever (6–10.6%), and night sweats (6–12.6%), as well as respiratory symptoms such as cough or dyspnea (6%) [1].

The most common sites of distant metastases include the lungs (34.6–71.0%), followed by bone (13.0–44.0%), lymph nodes (25%), liver (12.6–23.0%), and brain (5.1–16.0%) [1,5]. In agreement with these data, our patient presented with metastatic involvement of the lungs and subsequently developed bone metastases, reflecting the typical metastatic pattern and aggressive dissemination associated with sarcomatoid differentiation.

In patients with localized non-sarcomatoid renal cell carcinoma (RCC), nephrectomy is considered a potentially curative intervention. However, outcomes are significantly less favorable in cases of localized sarcomatoid RCC (sRCC) [26,27]. Despite surgical treatment with curative intent, approximately 77–80% of patients experience disease recurrence within 5–26 months. Moreover, these tumors are frequently bulky at presentation, often necessitating radical nephrectomy to achieve complete resection [27].

A substantial proportion of patients with sRCC—estimated at 60–80%—present with metastatic disease at diagnosis. In this setting, cytoreductive nephrectomy may be considered prior to the initiation of systemic therapy [1]. Retrospective studies in metastatic RCC have suggested a survival advantage for patients undergoing cytoreductive nephrectomy compared to those receiving systemic therapy alone [28,29].

Specifically, in a study including 189 patients with sRCC, median overall survival was 10.2 months in patients who underwent cytoreductive nephrectomy, compared to 5.5 months in those who did not [30]. Similarly, the largest epidemiological analysis of sRCC to date demonstrated a modest but measurable survival benefit in patients with good performance status undergoing nephrectomy [5]. In this cohort of 472 patients with metastatic sRCC, disease-specific survival at 1, 3, and 5 years was 33.7%, 10.8%, and 6.2%, respectively, in surgically treated patients, compared to 11.5%, 1.9%, and 0% in those managed non-surgically. Median disease-specific survival was 7 months (IQR 3–17) versus 4 months (IQR 2–7), respectively [5]. Furthermore, multivariate Cox regression analysis identified cytoreductive nephrectomy as an independent predictor of improved disease-specific survival (HR 0.53, 95% CI 0.43–0.66; *p* < 0.001) [5]. In our case, the patient initially underwent radical nephrectomy with curative intent; however, early metastatic progression was subsequently documented, consistent with the high recurrence rates and aggressive biological behavior described in sarcomatoid RCC.

Historically, systemic treatment options for sarcomatoid renal cell carcinoma were limited and associated with poor outcomes. Cytotoxic chemotherapy regimens, including doxorubicin-, ifosfamide-, and gemcitabine-based combinations, showed inconsistent activity, with low response rates, short progression-free survival, and median overall survival generally below one year. Although selected studies suggested that tumors with a high proportion of sarcomatoid differentiation might derive some benefit from chemotherapy, these findings were exploratory and not sufficient to establish chemotherapy as an effective standard approach. These limited results emphasized the need for more effective systemic therapies and provided the clinical background for the subsequent introduction of immune checkpoint inhibitor-based regimens in this aggressive RCC subtype [31,32,33,34,35].

### 3.2. Role of Immunotherapy

Immune checkpoint inhibitors (ICIs) have demonstrated the most significant therapeutic progress in the management of sarcomatoid renal cell carcinoma (sRCC), a subtype historically associated with poor response to conventional systemic therapies. This improved efficacy is thought to be related, at least in part, to the distinct immunogenic profile of sRCC.

Previous studies have shown that sRCCs exhibit higher expression of programmed death-ligand 1 (PD-L1) on tumor cells and increased infiltration with tumor-infiltrating lymphocytes (TILs) compared to non-sarcomatoid RCCs [36,37]. Concomitant expression of PD-L1 and TILs has been reported in up to 50% of sRCC cases, supporting the hypothesis that the PD-1/PD-L1 axis plays a key role in tumor immune evasion and represents a relevant therapeutic target [1].

These biological characteristics provide a rationale for the use of immune checkpoint blockade in sRCC and may explain the enhanced clinical responses observed in this subgroup. In particular, the combination of nivolumab and ipilimumab has shown superior outcomes compared to sunitinib in patients with sarcomatoid features, as demonstrated in post hoc analyses of the CheckMate 214 trial. Patients receiving combination immunotherapy exhibited higher objective response rates, improved progression-free survival, and prolonged overall survival. Furthermore, combination strategies involving ICIs and VEGF-targeted therapies have also demonstrated promising efficacy, with improved response rates and disease control compared to monotherapy approaches [1].

In the present case, the patient achieved an initial favorable radiological response following nivolumab plus ipilimumab therapy, consistent with the enhanced sensitivity of sRCC to immune checkpoint inhibition described in the literature. However, treatment discontinuation was required due to severe immune-related endocrine toxicity, highlighting the balance between therapeutic efficacy and treatment-related adverse events.

Nevertheless, although immunotherapy was discontinued in June 2025, another particular feature of this case is the patient’s subsequent clinical course in the context of the generally unfavorable prognosis of sarcomatoid renal cell carcinoma. Real-world data, such as those from the ARON-1 study, which included 350 patients with sRCC, reported a median overall survival of approximately 27 months in patients treated with first-line immune checkpoint inhibitor–based combinations, significantly shorter than that observed in patients without sarcomatoid differentiation (35.3 months; *p* = 0.013) [38].

In the same study, several factors were associated with a more favorable prognosis, including male sex, prior nephrectomy, and the absence of liver, bone, or lymph node metastases. In contrast, our patient presented with several adverse prognostic factors, including female sex, metastatic disease, and a high-grade sarcomatoid component, the latter having been reported as an independent predictor of mortality (HR 1.92) [38].

Despite this, the patient showed an initially favorable response to immunotherapy, and her survival beyond the expected interval, despite early treatment discontinuation, suggests the possibility of a durable immunological effect (“carry-over effect”) of immune checkpoint inhibitors. This phenomenon, described in the recent literature, reflects the persistence of antitumor immune activation even after treatment cessation and may explain the relatively favorable course observed in this case despite the presence of unfavorable prognostic factors.

### 3.3. Immune-Related Adverse Events Associated with Immunotherapy

Immune checkpoint inhibitors (ICIs), particularly the combination of nivolumab and ipilimumab, are associated with a broad spectrum of immune-related adverse events affecting multiple organ systems, including the endocrine, metabolic and nervous systems (irAEs). Overall, irAEs of any grade have been reported in up to 81% of patients with renal cell carcinoma, with dermatologic (49.8%), endocrine (33%), and gastrointestinal (29.6%) toxicities being the most frequently observed. Notably, treatment-related adverse events lead to therapy discontinuation in approximately 22% of cases, highlighting both their clinical significance and the need for effective management strategies [38].

Endocrine irAEs represent one of the most common and clinically relevant toxicities associated with ICIs. These may affect multiple endocrine organs, including the thyroid, pituitary, adrenal glands, and pancreas, and can range from mild dysfunction to life-threatening conditions [20,39]. Among these, thyroid dysfunction is the most frequently reported, occurring in 5.2–28% of patients receiving combination ICI therapy, with a higher incidence observed in dual checkpoint blockade compared to monotherapy [20,39]. Thyroiditis often evolves into permanent hypothyroidism requiring long-term hormone replacement.

In the present case, the patient developed severe endocrine toxicity, including autoimmune hypophysitis and hypothyroidism, leading to discontinuation of immunotherapy. This clinical course is consistent with the literature, where endocrine irAEs are frequently irreversible and require long-term management.

Adrenal insufficiency, although less common, is a potentially life-threatening complication, with reported incidence rates ranging from 4.7% to 10.2% in patients with RCC treated with nivolumab and ipilimumab [38,39,40,41,42]. Clinical presentation is often nonspecific, including fatigue and asthenia, which may delay diagnosis. Early recognition is essential, as most cases require lifelong glucocorticoid replacement.

Adrenal insufficiency was considered in the differential diagnosis, given the history of immune checkpoint inhibitor-related hypophysitis and the association of hypotension, hyponatremia, hyperkalemia and hypoglycemia at presentation. However, cortisol, ACTH and extended pituitary hormone testing were not available in our institution during hospitalization. Moreover, the metabolic abnormalities and renal dysfunction improved after fluid resuscitation, correction of electrolyte disturbances and supportive treatment, supporting a major contribution of dehydration, reduced oral intake, vomiting and prerenal acute kidney injury. Nevertheless, adrenal insufficiency could not be definitively excluded and the patient was advised to undergo prompt endocrinological reassessment after discharge, including cortisol, ACTH and pituitary hormone evaluation.

Similarly, ICI-induced type 1 diabetes mellitus is a rare but severe adverse event, with an estimated incidence of 0.2–1.4% [41,43,44]. Although not observed in our patient, this entity highlights the broad spectrum of endocrine toxicity associated with immune checkpoint blockade.

Beyond endocrine involvement, renal toxicity such as immune-mediated acute interstitial nephritis (AIN) has been reported in approximately 1.7% of patients with metastatic RCC receiving ICIs [45]. However, in our case, the acute kidney injury was most likely prerenal in mechanism, occurring in the setting of severe dehydration secondary to vomiting, with complete recovery of renal function following volume repletion by the time of discharge. The vomiting was, in turn, most likely caused by electrolyte disturbances rather than by a neurological or structural cause. Moreover, the presence of a solitary kidney, with secondary lesions documented on imaging, represented an additional factor of vulnerability for the rapid deterioration of renal function.

Importantly, emerging evidence suggests that the development of irAEs may be associated with improved oncologic outcomes, reflecting a heightened immune response that simultaneously targets tumor cells and normal tissues. This association has also been described in metastatic RCC [44]. In our patient, the occurrence of severe endocrine toxicity followed an initially favorable response to immunotherapy, supporting this hypothesis.

The management of irAEs remains challenging, particularly in cases involving multiple organ systems. Current guidelines recommend permanent discontinuation of ICIs in patients who develop severe (grade 4) toxicities [12,46]. However, recent data suggest that re-challenge may be feasible in selected cases, with recurrence rates of approximately 28.8% [47,48]. This raises an important clinical dilemma regarding the balance between therapeutic benefit and toxicity, particularly in patients with an initial favorable response.

The spectrum of immune-related adverse events associated with immune checkpoint inhibitors, particularly endocrine toxicities, is summarized in Table 2. As illustrated, these adverse events are relatively frequent, may involve multiple organ systems, and can range from mild to life-threatening conditions. Overall, our case underscores the importance of early recognition, multidisciplinary management, and careful monitoring of endocrine and metabolic complications in patients receiving immune checkpoint inhibitors.

Beyond endocrine involvement, immune-related adverse events may also affect the nervous system, although less frequently. Although endocrine toxicities are among the most commonly reported irAEs, neurological complications, while rare, can be severe and potentially life-threatening.

Immune checkpoint inhibitor-induced myasthenia gravis is a well-recognized but uncommon entity, typically presenting with progressive or fluctuating muscle weakness, often with a proximal predominance. Clinical manifestations frequently include bulbar involvement, such as ptosis, diplopia, extraocular movement abnormalities, dysphagia, and facial muscle weakness, and may also involve respiratory muscles in severe cases [49]. The condition usually develops within the first two months after initiation of immune checkpoint inhibitor therapy, with a median onset of approximately six weeks (range 2–12 weeks) [50,51]. Most reported cases represent de novo presentations (72.7%), while others correspond to exacerbations of previously diagnosed (18.2%) or subclinical myasthenia gravis (9.1%) [51].

In our case, the development of new-onset severe generalized muscle weakness with loss of ambulation raised suspicion for an immune-related neuromuscular disorder. Although the diagnosis has not yet been confirmed, this clinical presentation highlights the importance of considering neurological irAEs in patients with prior exposure to immune checkpoint inhibitors, particularly in the setting of multisystem involvement.

In the present case, the persistence of severe muscle weakness after correction of hyperkalemia raised suspicion of a possible immune-related neuromuscular adverse event. However, this diagnosis could not be confirmed because specific antibody testing and neurophysiological investigations were not available during hospitalization. Although outpatient ENMG and further neurological assessment were recommended, these investigations could not be completed because of subsequent clinical deterioration. Therefore, the possibility of immune checkpoint inhibitor-related myasthenia gravis should be interpreted as a clinical suspicion and differential diagnostic consideration, rather than a confirmed immune-related adverse event.

In addition to endocrine dysfunction, patients receiving systemic oncologic therapy are at increased risk of significant metabolic disturbances, often with a multifactorial etiology. In our case, the patient developed severe hyperkalemia in the setting of repeated vomiting and dehydration. This was most likely caused by volume depletion, leading to reduced renal perfusion and impaired potassium excretion, possibly accompanied by a degree of hemoconcentration and prerenal acute kidney injury. The rapid correction of serum potassium levels after volume repletion supports a functional, volume-dependent mechanism rather than a primary endocrine cause or structural renal impairment. This interpretation is further supported by the marked improvement in renal function, with the estimated glomerular filtration rate increasing from 45.4 mL/min/1.73 m^2^ at admission to 77.9 mL/min/1.73 m^2^ at discharge.

Moreover, the presence of a solitary functioning kidney in this patient, with secondary lesions documented on imaging, could initially have suggested structural renal impairment as a mechanism of acute kidney injury. However, the complete recovery of renal function following fluid and electrolyte rebalancing supports the predominantly functional, prerenal nature of the impairment, while also identifying this as an additional risk factor for rapid clinical deterioration in the setting of volume depletion.

Although pazopanib had been administered as second-line therapy after disease progression, it had been discontinued approximately two weeks before the January 2026 admission. Therefore, while recent exposure to pazopanib was part of the patient’s oncologic background, a direct contribution to the acute dehydration, electrolyte disturbances, and persistent muscle weakness at admission was considered less likely than the combined effects of vomiting, reduced oral intake, prerenal acute kidney injury, endocrine vulnerability, and concomitant chronic cardiovascular medication.

In addition, concomitant chronic medication may have contributed to the development or worsening of electrolyte imbalance and renal dysfunction. The patient was receiving sacubitril/valsartan and dapagliflozin before admission. Sacubitril/valsartan, an angiotensin receptor–neprilysin inhibitor, is an established therapy for chronic heart failure, with proven benefits in reducing cardiovascular mortality and heart failure hospitalization. However, because of its valsartan component and interference with the renin–angiotensin–aldosterone system, it may contribute to hyperkalemia, particularly in vulnerable patients with impaired renal perfusion, reduced effective circulating volume, or pre-existing renal dysfunction [52]. Dapagliflozin, a sodium–glucose cotransporter 2 inhibitor, has demonstrated cardiovascular and renal benefits and is widely used in patients with heart failure and chronic kidney disease. Nevertheless, through its osmotic diuretic effect, it may contribute to volume depletion, especially in patients with reduced oral intake, vomiting, or acute illness [53]. In the present case, the combination of vomiting, dehydration, prerenal acute kidney injury, solitary kidney status, renal metastatic involvement and concomitant therapy with sacubitril/valsartan and dapagliflozin likely created a multifactorial predisposition to hyperkalemia and renal dysfunction. Therefore, the electrolyte abnormalities should not be interpreted as the consequence of a single mechanism, but rather as the result of overlapping oncologic, renal, metabolic and pharmacological factors.

Another mechanism that should be considered in the context of this clinical presentation is the neuromuscular effects of hyperkalemia. Severe hyperkalemia, usually above 7 mmol/L, may cause flaccid paralysis even in patients without pre-existing neuromuscular disease. In a systematic review including 119 patients with hyperkalemia-induced paralysis, most presented with flaccid paralysis or severe muscle weakness at mean serum potassium levels of approximately 8.8 mEq/L, and symptoms resolved completely in 87% of cases after correction of the electrolyte disturbance [54].

In our case, the severe muscle weakness with loss of ambulation initially raised suspicion of an immune-mediated neuromuscular disorder. However, severe hyperkalemia could not be excluded as a possible contributing etiology, given its potentially reversible nature.

Myalgia is less commonly encountered as a direct manifestation of hyperkalemia, but it may occur in the context of acute rhabdomyolysis, which in turn can lead to hyperkalemia through potassium release from damaged muscle fibers. In such situations, the clinical picture is typically characterized by the sudden onset of muscle pain, swelling, and weakness, associated with marked creatine kinase elevation and myoglobinuria [55]. In the present case, the absence of biological markers suggestive of rhabdomyolysis makes this hypothesis unlikely, although it was considered in the differential diagnosis. This combination of endocrine, metabolic, and possibly neuromuscular involvement suggests a pattern of extensive immune dysregulation induced by immune checkpoint blockade.

Overall, our case underscores the importance of early recognition, multidisciplinary management, and careful monitoring of both endocrine and metabolic complications in patients receiving immune checkpoint inhibitors. Importantly, this case also illustrates the diagnostic challenge of distinguishing immune-related adverse events from more common causes of metabolic imbalance, such as gastrointestinal losses, particularly in patients with complex oncologic and endocrine backgrounds.

## 4. Limitations

This report has several limitations. First, it describes a single case, and the findings cannot be generalized to all patients with metastatic renal cell carcinoma. Second, this article is not a systematic review, and the literature was not selected according to predefined criteria. Third, several diagnostic assessments could not be completed, including the exact quantification of the sarcomatoid component, extended endocrine testing and confirmatory neurophysiological evaluation. Despite these limitations, this case provides clinically relevant insights into the complex interplay between oncologic treatment, immune-related adverse events, and metabolic complications, highlighting the importance of multidisciplinary evaluation and close monitoring in such patients.

## 5. Conclusions

This case highlights the complex interplay between advanced sarcomatoid renal cell carcinoma, systemic oncologic therapy, and multisystem immune-related and metabolic complications. While immune checkpoint inhibitors have significantly improved outcomes in patients with metastatic disease, they are associated with a broad spectrum of adverse events that may involve multiple organ systems and complicate clinical management.

Our case illustrates that endocrine toxicity, metabolic disturbances, and possible neurological involvement may coexist, creating diagnostic and therapeutic challenges. In particular, electrolyte imbalances such as hyperkalemia may result from multifactorial mechanisms, including gastrointestinal losses and volume depletion, rather than direct treatment-related toxicity alone.

Early recognition of these complications, careful differential diagnosis, and a multidisciplinary approach are essential to prevent clinical deterioration and to optimize patient outcomes. Further studies are needed to better characterize the relationship between immune-related adverse events and treatment response, as well as to guide management strategies in patients with complex, multisystem involvement.

## Figures and Tables

**Table 1:** Relevant Laboratory Parameters.

Parameter	Patient Value	Reference Range
**Hemoglobin (Hb)**	10.1	13.5–17.5 g/dL (male)
**Leukocytes (WBC)**	3.63	4.0–10.0 × 10^9^/L
**Neutrophils**	3.23	1.5–8 × 10^9^/L
**Lymphocytes**	0.34	1.5–4.0 × 10^9^/L
**Platelets (PLT)**	120	150–400 × 10^9^/L
**AST (SGOT)**	16	0–50 U/L
**ALT (SGPT)**	7	0–50 U/L
**Total Bilirubin**	0.8	0.1–1.2 mg/dL
**Serum Albumin**	3.96	3.5–5.2 g/dL
**Ferritin**	1895	15–150 ug/L
**CRP**	35.96	<5 mg/L
**Urea**	128.5	16.6–48.5 mg/dL
**Serum Creatinine**	1.27	0.7–1.2 mg/dL
**eGFR (CKD-EPI)**	48	>90 mL/min/1.73 m^2^
**K**	6.3	3.5–5.1 mEq/L
**Na**	133	136–145 mEq/L
**Mioglobin**	238	0–107 ng/mL
**D-dimers**	1490	<500 ng/mL FEU
**FT4**	1.78	0.7–1.48 ng/dL
**TSH**	0.0487	0.35–4.94 uUi/mL

**Table 2:** Immune-related adverse events associated with immune checkpoint inhibitors in RCC and correlation with the present case.

Adverse Event	Reported Incidence	Clinical Characteristics	Management	Relevance to Present Case
Any-grade irAEs	~81%	Multisystem involvement; most commonly dermatologic, endocrine, and gastrointestinal	Symptomatic treatment, monitoring, possible therapy interruption	Severe irAEs contributed to treatment discontinuation
Endocrine toxicities (overall)	~33%	Involvement of thyroid, pituitary, adrenal glands; may be severe or irreversible	Hormone replacement, endocrine evaluation	Clinically relevant; hypophysitis and thyroid dysfunction were documented
Thyroid dysfunction	5.2–28%	Thyroiditis progressing to hypothyroidism; may occur weeks to months after ICI initiation	Long-term levothyroxine replacement	Present; required long-term hormone replacement
Hypophysitis	Not precisely quantified (more frequent with combination therapy)	Pituitary dysfunction with multiple hormonal deficiencies	Corticosteroids, hormone replacement	Previously diagnosed after ICI therapy
Adrenal insufficiency	4.7–10.2% (RCC)	Nonspecific symptoms (fatigue, low energy), potentially life-threatening	Long-term glucocorticoid replacement	Considered in the differential diagnosis; not confirmed
Type 1 diabetes mellitus	0.2–1.4%	Acute onset hyperglycemia, often within first 3 months	Insulin therapy	Not observed
Acute interstitial nephritis	~1.7%	Acute kidney injury, often difficult to differentiate from other causes	ICI discontinuation, corticosteroids	Unlikely; AKI was predominantly prerenal
irAEs and treatment response	—	Increased incidence of irAEs associated with improved therapeutic response	Not applicable	Initial favorable oncologic response occurred before toxicity-related discontinuation
irAE-related treatment discontinuation	~22%	Severe adverse events requiring cessation of therapy	Discontinuation or possible rechallenge	Immunotherapy was discontinued because of severe endocrine toxicity

Data synthesized from previously published studies on immune-related adverse events in RCC and integrated with findings from the present case [39,40,41,42,43,44,45,46,47,48,49,50].

## Data Availability

All data used in this study are from previously published sources, as cited in the reference list. No new data was generated.

## Abbreviations

The following abbreviations are used in this manuscript:

AIN	Acute interstitial nephritis
ASCO	American Society of Clinical Oncology
ccRCC	Clear cell renal cell carcinoma
CT	Computed tomography
CTLA-4	Cytotoxic T-lymphocyte-associated antigen 4
eGFR	Estimated glomerular filtration rate
ENMG	Electroneuromyography
ESMO	European Society for Medical Oncology
ICI	Immune checkpoint inhibitor
IMDC	International Metastatic Renal Cell Carcinoma Database Consortium
irAE	Immune-related adverse event
LVEF	Left ventricular ejection fraction
mRCC	Metastatic renal cell carcinoma
NCCN	National Comprehensive Cancer Network
NYHA	New York Heart Association
ORR	Overall response rate
OS	Overall survival
PD-1	Programmed cell death protein 1
PD-L1	Programmed death-ligand 1
PFS	Progression-free survival
RCC	Renal cell carcinoma
sRCC	Sarcomatoid renal cell carcinoma
TILs	Tumor-infiltrating lymphocytes
VEGF	Vascular endothelial growth factor
WHO	World Health Organization
